# Benefits of martial arts on the functional capacity of elderly people: a systematic review and meta-analysis

**DOI:** 10.1016/j.clinsp.2025.100830

**Published:** 2025-11-17

**Authors:** Dário Lucas Costa de Mendonça, José Matos Raider Junior, Leandro Bruno Barbosa da Silva, Angelica Castilho Alonso, Júlia Maria D'Andrea Greve, Luiz Eugênio Garcez-Leme

**Affiliations:** a Orthogeriatrics Group, Institute of Orthopedics and Traumatology, Hospital das Clinicas da Faculdade de Medicina da Universidade de Sao Paulo (HC-FMUSP), Sao Paulo, SP, Brazil; b Movement Studies Laboratory (LEM), Faculdade de Medicina da Universidade de Sao Paulo (FMUSP), Sao Paulo, SP, Brazil; c Postgraduate Program in Rehabilitation Sciences, Universidade Nove de Julho (UNINOVE), Sao Paulo, SP, Brazil; d Postgraduate Program in Aging Sciences, Universidade Sao Judas Tadeu (USJT), Sao Paulo, SP, Brazil

**Keywords:** Martial arts, Aged, Balance, Muscle strength, Quality of life

## Abstract

**Objectives:**

To evaluate the benefits of martial arts on the functional aspects and quality of life of elderly individuals, and the level of evidence of the results.

**Methods:**

A systematic review of randomized clinical studies was performed using the Cochrane, PubMed, Embase, CINAHL, SciELO, LILACS and PEDro electronic databases. A specific search strategy was developed for each database, and all published studies were included, regardless of the data collection instruments or the date of publication. The eligibility criteria followed the PICOT strategy, and the level of evidence of all outcomes was assessed using the GRADE scale. Initially, 1682 studies were retrieved, and duplicates were identified and excluded using the RAYYAN platform. After analyzing the title and abstract, eligible studies were read in full, and ten studies were selected for inclusion in this study.

**Results:**

The meta-analysis showed a significant difference in the assessment of functional mobility for the martial arts group, whereas the control group (physically active) showed a difference in balance and handgrip strength. Quality of life meta-analysis was not possible because of study heterogeneity. In the evaluation of the level of evidence, GRADE results indicated a serious risk of bias because there was no blinding of the raters and a serious risk of imprecision because of the low number of patients evaluated; thus, the evidence has low clinical certainty.

**Conclusion:**

Taekwondo (adapted) and Muay Thai (ritual dance) are indicated as intervention approaches to improve the functional mobility of elderly people. However, the results of related studies have a low level of evidence. Future studies may change the state of the art.

**Registration:**

The review protocol was registered on PROSPERO (ID number and hyperlink: CRD42022313588).

## Highlights

• Taekwondo and Muay Thai reduce the risk of falls in elderly individuals.
• Physical exercises are more effective than martial arts for improving handgrip strength.
• Quality of life is improved by martial arts, but meta-analysis was not possible because of study heterogeneity.

## Introduction

Living longer is one of the greatest achievements of humankind, but a longer life, in and of itself, is not sufficient; it is necessary to live with a quality of life.^1^ Adding satisfaction to years lived is the most appropriate construct of successful ageing because it takes into account quantitative data, i.e., years lived, and qualitative data, i.e., the increased perception of values and goals achieved.^2^

In contrast, with advancing age, the occurrence of falls becomes more prevalent, and falls directly decrease the quality of life and life expectancy of elderly individuals. Individuals in this age group commonly report a fear of falling, affecting the confidence and autonomy of the individual, thus decreasing exposure to physical activities.^3^ Within this context, the World Health Organization recommends active ageing through regular physical activity because it confers several benefits, such as a decreased mortality rate. These activities can be performed as part of recreation and leisure (playing, games, sports, or planned exercise), through locomotion (walking or cycling), and while performing work tasks or completing household chores.^4^

Thus, body practices can be used as intervention strategies because they are “individual or collective expressions of body movement, arising from knowledge and experience around games, dance, sports, wrestling, or gymnastics, constructed in a systematic (at school) or unsystematic (free time/leisure)” way.^5^ These are culturally relevant physical practices that concern humans in motion, who express themselves bodily through gestures full of values and meanings.

Martial arts are body practices that use attack and self-defence techniques and have as objectives physical and mental improvements in individuals.^6^ Therefore, it is important to critically analyze the state of the art so that health professionals can understand the therapeutic effects of martial arts in elderly people and indicate its practice as a strategy for successful ageing. Thus, the objectives of this study were to evaluate the benefits of martial arts with regard to the quality of life and functional aspects of elderly individuals and to analyse the level of evidence of the results.

## Methods

### Type of study

This was a systematic review registered in the International Prospective Register of Systematic Reviews (PROSPERO) (number: CRD42022313588). The eligibility criteria followed the Patient, Intervention, Comparison, Outcome, Time (PICOT) strategy as indicated by PRISMA^7^ (Table 1). The PICOT question was as follows: Compared with elderly individuals in any control group, do elderly patients undergoing martial arts interventions have a lower risk of falls and better quality of life at any time point evaluated after the intervention, based on the results for specific outcomes (balance, fear of falling, fall event, muscle strength, and quality of life)?

**Table 1:** PICOT strategy.

Acronym	Description
P	Elderly individuals
I	Martial arts practices
C	Any control group
O	Balance; fear of falling; fall event; muscle strength; quality of life.
T	At any time, point assessed after the intervention.

P, Patient; I, Intervention; C, Comparison; O, Outcome; T, Time.

### Procedures

All published clinical trials, regardless of the data collection instruments or the date of publication, were included. Clinical trials that dealt exclusively with tai chi were excluded because there are already several systematic reviews regarding its clinical efficacy.^8^, ^9^, ^10^, ^11^, ^12^, ^13^, ^14^ Studies that addressed patients with Parkinson’s disease with locomotor system impairment, thus preventing their comparison with other groups, and studies without a control group were excluded.

The CENTRAL (Cochrane), PubMed, Embase, CINAHL and PEDro electronic databases were searched using strategies specific to each database. The search strategy was constructed using as a reference Cochrane reviews^13^^,^^15^ with variations of MeSH keywords: martial arts, elderly, and gerontology. Duplicate articles were identified using EndNote and excluded.

**Fig. 1:**
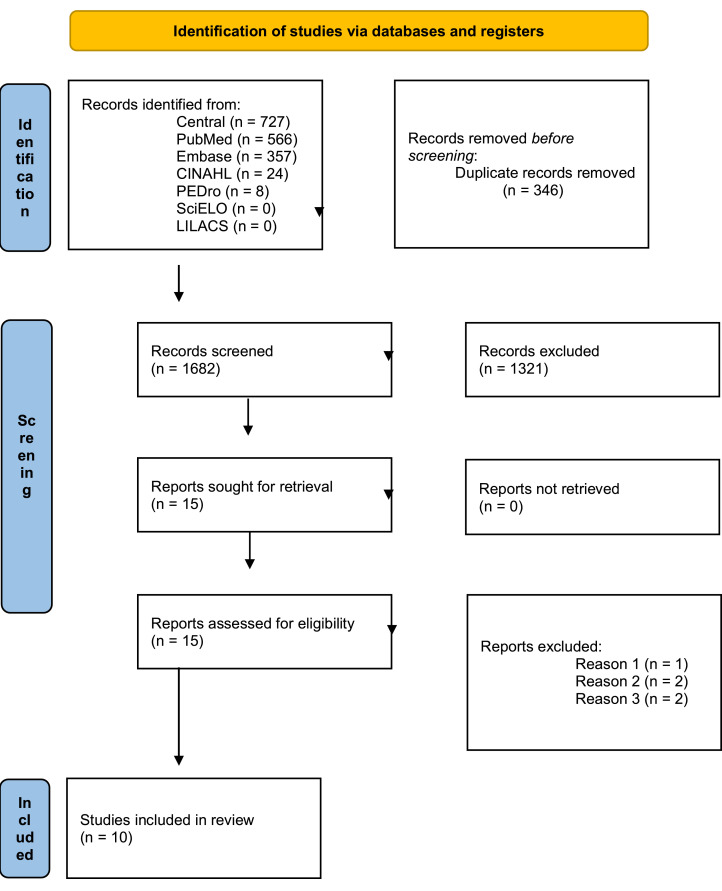
PRISMA 2020 flow diagram for new systematic reviews which included searches of databases and registers only. Legend: Reason 1 ‒ Participants younger than 60-years of age, Reason 2 ‒ Participants had severe functional impairment due to Parkinson’s disease, and Reason 3 ‒ The study did not have a control group. From: Page MJ, McKenzie JE, Bossuyt PM, Boutron I, Hoffmann TC, Mulrow CD, et al. The PRISMA 2020 statement: an updated guideline for reporting systematic reviews. BMJ 2021;372:n71. doi:10.1136/bmj.n71.

The articles were selected by three independent examiners (DLCM, JMRJ and LBBS) based on reading the title or abstract, and cases of disagreement were decided by consensus after discussion. In the absence of consensus, a fourth reviewer (LEGL) was consulted. Then, the potentially eligible articles were read in full, and the reference lists of all selected articles were consulted with the purpose of finding new publications for this review. The last active search occurred in July 2025. The article retrieval and selection process is shown in the flowchart in Fig. 1.

Initially, 1682 articles were identified in the databases consulted: 727 in CENTRAL (Cochrane), 566 in PubMed, 357 in Embase, 24 in CINAHL and 8 in PEDro. No eligible studies were found in the SciELO and LILACS databases. A total of 347 duplicates were identified using EndNote and excluded. A further 1319 articles were excluded after reading the title and, in cases of doubt, after reading the abstract. Thus, 16 articles were selected for full-text reading and analysis. After analysing the studies, one article was excluded because the participants were not elderly, two articles were excluded because the participants were diagnosed with Parkinson’s disease, and two articles were excluded because they did not have a control group. Thus, ten eligible articles were selected that included elderly individuals and evaluated the proposed outcomes, regardless of the time of analysis (Table 2).

**Table 2:** Characteristics of the eligible studies.

Author, year	Groups (N)	Frequency and duration	Outcome measures	Key findings
Valdés-Badilla, 2024	Taekwondo (13) Multicomponent (12) Walking (12) Control (14)	16 weeks, 60 min three times a week	Blood pressure, morphological variables, frequency of food consumption, cognitive status, health-related quality of life, physical fitness, and postural balance.	↑ Taekwondo and Multicomponent improved more than Control in TUG, handgrip strength, and postural balance.
Valdés-Badilla, 2023	Taekwondo (14) Multicomponent (11)	8 weeks, 60 min three times a week	Blood pressure, morphological variables, frequency of food consumption, health-related quality of life (HRQoL), physical fitness, handgrip strength, and postural balance.	↑ HRQoL in mental and general health. Chair stand, arm curl, 2-min step, chair sit-and-reach tests. Postural balance.
Kujach, 2022	Judo (20) Control (20)	12 weeks, 45 min three times a week	Body composition, cognitive function, peripheral brain-derived neurotrophic factor concentration (BDNF), and muscle function (postural control and muscle strength).	↑ BDNF and muscle function
Areeudomwong, 2019	Muay Thai (39) Control (39)	Four weeks, 80 min, reassessed after 16 weeks	Dynamic balance performance, static balance performance; lower limb muscle strength (push-pull dynamometer); back and leg flexibility (chair sit and reach test); and agility (8-foot Up and Go test).	↑ TUG, Romberg test (eyes open), muscle strength, chair sit and reach test, 8-foot Up and Go test
Ciaccioni, 2019	Judo (16) Control (14)	16 weeks, one hour every two weeks	Body mass index, waist-hip circumferences, upper-lower body flexibility, strength and coordination, perceived physical and mental health, body image, fear of falling.	↑ Upper and lower body flexibility and strength. ↓ Hip circumferences
Su-Youn Cho, 2019	Taekwondo (19) Control (18)	16 weeks, one hour five times a week	Senior Fitness Test. Brain-derived neurotrophic factor (BDNF), vascular endothelial growth factor (VEGF), insulin-like growth factor-1 (IGF-1). Systolic-diastolic, blood flow velocity and pulsatility index of the middle cerebral artery. Mini-Mental State Examination for dementia screening; Stroop Color and Word Test	↑ Lower body strength and flexibility, aerobic endurance levels, BDNF, VEGF, IGF-1 serum levels, and colour-word test scores
Janyacharoen, 2018	Muay Thai (28) Control (28)	12 weeks, 40 min three times a week	Physical functions and quality of life.	↑ Physical function and quality of life
Lip, 2015	Ving Tsun (12) Control (27)	12 weeks, one hour per week	Bone strength of the distal radius; muscular strength in the limbs; shoulder joint mobility; and balance performance and self-efficacy.	No significant differences
Youm, 2011	Taekwondo (10) Walking (10) Control (10)	12 weeks, one hour three times a week	Double-leg balance control (force platform, AMTI OR6–7, Watertown, MA, US).	↑ Balance control
Cromwell, 2007	Taekwondo (20) Control (20)	11 weeks, one hour twice a week	Balance and walking ability (single-leg stance - SLS, Multidirectional Reach Test - MDRT, Timed Up and Go - TUG, walking velocity, cadence, gait stability ratio - GSR, and sit-and-reach - S&R).	↑ MDRT (backwards, right, and left), TUG, walking velocity, GSR, and S&R

N, Sample size.

### Quality of the studies

The tool for the assessment of study quality and reporting in exercise (TESTEX) was used to assess the methodological quality of the randomized clinical trials and thus determine their level of bias. This scale was chosen because it evaluates clinical trials of physical exercise interventions, and as one of the quality criteria of other scales is the blinding of the participant and the researcher, its application with the type of intervention studied herein is more appropriate. Thus, the TESTEX is suitable for the present study.^16^

The Grading of Recommendations Assessment, Development and Evaluation (GRADE) system was used to assess the level of evidence and thus classify the strength of evidence. This system is used to evaluate outcomes individually, and the results yield recommendations that help specialists in clinical decision-making.^17^

## Results

**Fig. 2:**
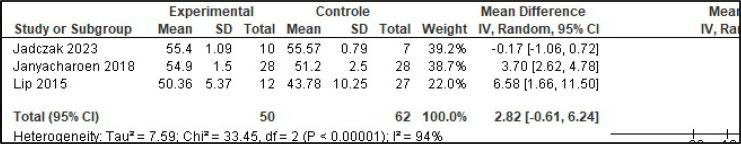
Meta-analysis of the assessment of balance (Berg Balance Scale).

**Fig. 3:**
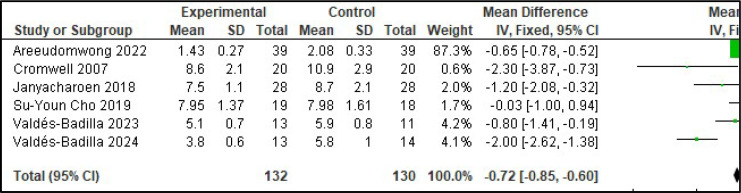
Meta-analysis of the assessment of functional mobility (Timed Up and Go Test).

**Fig. 4:**
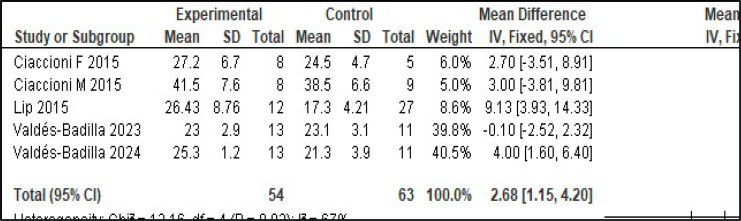
Meta-analysis of the assessment of handgrip strength.

It was not possible to perform a meta-analysis of quality of life with the eligible studies. However, it was possible to perform a meta-analysis of the assessment of postural balance^18^^,^^19^ (Fig. 2), functional mobility^18^^,^^20^, ^21^, ^22^^,^^24^^,^^25^ (Fig. 3), and handgrip strength^19^^,^^23^, ^24^, ^25^ (Fig. 4).

To assess the risk of falls, a meta-analysis of postural balance (Berg Balance Scale ‒ BBS) and functional mobility (Timed Up and Go Test ‒ TUG) was performed. The control group showed statistical superiority in the assessment of balance, but the martial arts group showed statistical superiority in the assessment of functional mobility [BBS ‒ heterogeneity: Tau² = 0.85; Chi² = 1.26, df = 1 (*p* = 0.26); I² = 20 %; global effect test: *Z* = 4.12 (*p* < 0.0001). TUG ‒ heterogeneity: Chi² = 24.65, df = 5 (*p* = 0.0002); I² = 80 %; test for overall effect: *Z* = 11.33 (*p* < 0.00001)]. To assess muscle strength, a meta-analysis of handgrip strength was performed, and the control group showed statistical superiority [heterogeneity: Chi² = 12.16, df = 4 (*p* = 0.02); I² = 67 %; test for overall effect: *Z* = 3.44 (*p* = 0.0006)].

Second, in the evaluation of the methodological quality of the studies (TESTEX; score 0/12) (Table 3), the studies showed fair methodological quality: Janyacharoen, 5^18^; Lip, 3^19^;; Areeudomwong, 5^20^; Su-Youn Cho, 6^21^; Cromwell, 5^22^; Ciaccioni, 4^23^; Youm, 4^26^; and Kujach, 5.^27^ However, two studies exhibited high quality: Valdés-Badilla, 10.^24^^,^^25^

**Table 3:** Evaluation of the methodological quality of the randomized clinical trials (TESTEX).

Author, year	Specification of inclusion criteria	Random allocation	Confidentiality in allocation	Similarity of groups at baseline or baseline	Evaluator masking in at least one outcome	Measurement of at least one primary outcome in 85 % of subjects	Intention to treat analysis	Comparison between groups in at least one primary outcome	Reporting of measures of variability for all outcome measures	Monitoring of the activities of the control group	Constancy of the relative intensity of the exercise	Characteristics of exercise volume and energy expenditure	Risk of bias (0/12)
Valdés-Badilla, 2024	1	1	1	1	1	1	0	1	0	1	1	1	10
Valdés-Badilla, 2023	1	1	1	1	1	1	0	1	0	1	1	1	10
Kujach, 2022	1	1	0	1	0	1	0	1	0	0	0	0	5
Areeudomwong, 2019	1	1	0	1	0	1	0	1	0	0	0	0	5
Ciaccioni, 2019	1	0	0	1	0	1	0	1	0	0	0	0	4
Su-Youn cho, 2019	1	1	1	1	0	1	0	1	0	0	0	0	6
Janyacharoen, 2018	1	1	0	1	0	1	0	1	0	0	0	0	5
Lip, 2015	1	0	0	0	0	1	0	1	0	0	0	0	3
Youm, 2011	1	1	0	0	0	1	0	1	0	0	0	0	4
Cromwell, 2007	1	1	1	0	0	1	0	1	0	0	0	0	5

Legend: 1 — yes; 0 — no; Risk of bias — the higher the score, the lower is the risk of bias.

The methodological control in the studies directly influenced the quality of evidence on the outcomes. In the evaluation of the level of evidence of outcomes (GRADE system) (Table 4), all outcomes exhibited low clinical certainty, severe risk of bias due to nonblinding of raters, and severe data imprecision due to the low number of participants assessed.

**Table 4:** GRADE evaluation of the methodological quality of the primary and secondary outcomes.

Evaluation of certainty							Nº of patients		Effect		Certainty	Importance
Nº of studies	Study design	Risk of bias	Inconsistency	Indirect evidence	Inaccuracy	Other considerations	Experimental	Control	Relative (95 % CI)	Absolute (95 % CI)		
Balance ‒ Berg Balance Scale - BBS (follow-up: mean 4-months)												
2	Randomized clinical trials	Severe (a)	Not serious	Not serious	Severe (b)	None	40	55	‒	MD 2.82 higher (0.61 lower to 6.24 higher)	⨁⨁◯◯ Low	Important
Functional mobility ‒ Timed Up and Go Test ‒ TUG (follow-up: mean 4-months)												
4	Randomized clinical trials	Severe (a)	Not serious	Not serious	Severe (b)	None	132	130	‒	MD 0.72 lower (0.85 lower for 0.6 minor)	⨁⨁◯◯ Low	Important
Handgrip strength ‒ Handgrip (follow-up: mean 4-months)												
4	Randomized clinical trials	Severe (a)	Not serious	Not serious	Severe (b)	None	54	63	‒	2.68 highest MD (1.15 highest to 4.2 highest)	⨁⨁◯◯ Low	Important

CI, Confidence interval; MD, Mean difference; severe (a) No blinding of the evaluators; severe (b) Total number of participants in the comparison is below the optimal information size.

## Discussion

When analysing the general state of the art of martial arts interventions for elderly people, it was possible to observe that there is a lack of studies on the subject and that, according to TESTEX scores, most studies have low methodological control due to challenges with regard to masking the interventions, thus increasing the level of bias in their results. The low number of participants in the groups also reflects negatively on GRADE scores because it makes it difficult to extrapolate the results to the external population. The results with a low level of evidence indicate low confidence in the effect; however, the results of future studies should have a significant impact on the estimation of the effect.^28^

### Risk of falls

Taking into account that the event with the highest risk of falling is a dynamic task, it is necessary to have good reach and functional mobility to be able to maintain balance during motor performance. Therefore, it is understood that having good functional control decreases the risk of falls.^29^ Thus, when analysing the meta-analysis of studies that evaluated the functional mobility of the elderly who received a martial arts intervention, the results showed significant improvement in the intervention group compared to the control group.^18^^,^^20^, ^21^, ^22^^,^^24^^,^^25^ Janyachoren^18^ and Areeudomwong^20^ evaluated the effect of interventions involving the ancient ritual dance of Thai boxing, Muay Thai. Cho^21^ and Cromwell^22^ evaluated the effect of interventions involving Taekwondo, and Valdés-Badilla adapted Taekwondo.^24^^,^^25^ In a systematic review conducted to evaluate the effect of Tai Chi in frail older adults and those with sarcopenia, older adults who received the intervention showed better performance on mobility tests, sitting down and standing up from a chair in 30-seconds, and physical activity, with a decrease in the number of falls and fear of falling.^30^

However, the results of the meta-analysis of the studies that used the BBS (Berg Balance Scale) showed significant improvement in the control group compared to the ancient Thai boxing group^18^ and compared to the Ving Tsun group.^19^ Among the studies analyzed, Janyachoren^18^ reported a significant result for martial arts in the TUG test and a significant result for the control group in the BBS evaluation. This result was not expected because in another systematic review that evaluated balance,^31^ a significant result in the TUG test was followed by a significant result in the BBS. The low methodological quality of the studies analyzed, as determined using the TESTEX (Table 2), may be the reason for the divergence in the results found.

### Muscle strength

The results of the meta-analysis of the evaluation of handgrip strength showed significant improvement in the physically active control group compared to the intervention group.^19^^,^^23^, ^24^, ^25^ Lip^19^ studied the effect of one hour of Ving Tsun training per week for 12-weeks, Ciaccioni^23^ studied the effect of one hour of Judo every 15-days for 16-weeks, Valdés-Badilla^24^ studied the effect of one hour of adapted Taekwondo three times a week for 16-weeks and for eight weeks.^25^ Handgrip strength is an important marker of general health status because a decline in this index is associated with decreased cognition, mobility, and functional status and increased mortality in elderly individuals.^32^, ^33^, ^34^ Thus, the result indicates that the practice time used was not sufficient to generate a significant difference from the physically active control group.

### Quality of life

In the present study, only one randomized clinical study was identified that used the WHOQOL-BREF,^18^ and one research group used the SF-36^24^^,^^25^ to assess the quality of life of participants, thus making it impossible to perform a meta-analysis. The Muay Thai ritual dance study^18^ showed that 12-weeks of practice improved the quality of life of elderly participants in the physical and psychological domains. The adapted Taekwondo studies.^24^^,^^25^ showed that eight weeks of practice increased the mental health and general health dimensions, and 16-weeks improved the body pain and general health dimensions. The results are consistent with those of a study in which a higher level of physical activity was associated with a better perception of quality of life among elderly people with different health conditions.^35^ A similar result was found in an observational study in which the short version of a quality-of-life questionnaire (WHOQOL-BREF) and a specific version for the elderly (WHOQOL-old) were used. In that study, compared to physically active elderly people, elderly Kendo practitioners reported better quality of life, especially in the physical domain but also in the environment domain, in addition to the social participation and past, present and future activity domains.^36^

## Conclusion

Compared with control interventions, interventions involving Taekwondo (adapted) and Muay Thai (ritual dance) led to significant differences and, therefore, should be considered as approaches to improve the functional mobility of elderly individuals. The state of the art of martial arts interventions reveals that there is little academic production regarding martial arts interventions and successful ageing, and that the results have a low level of evidence. Thus, future studies with better methodological control may modify the results obtained in this study.

## Authors’ contributions

Mendonça DLC was responsible for the study design, data collection, data analysis and preparation of the manuscript; Raider Junior JM was responsible for the study design, data collection, data analysis and critical review of the manuscript; Silva LBB was responsible for the data collection and data analysis; Alonso AC was responsible for the study design and critical review of the manuscript; Greve JMD was responsible for the study design and critical review of the manuscript; and Garcez-Leme LE supervised the study and was responsible for the study design and critical review of the manuscript.

## Funding

There was no funding for the preparation, execution, or submission of the study.

## Conflicts of interest

The authors declare no conflicts of interest.

## Data Availability

The datasets generated during and/or analysed during the current study are available from the corresponding author on reasonable request.
